# Antiproliferative Effect of Inorganic and Organic Selenium Compounds in Breast Cell Lines

**DOI:** 10.3390/biomedicines11051346

**Published:** 2023-05-03

**Authors:** Nayara Souza da Costa, Luíza Siqueira Lima, Franciele Aparecida Mendes Oliveira, Maria Eduarda Andrade Galiciolli, Mariana Inocêncio Manzano, Quelen Iane Garlet, Ana Carolina Irioda, Cláudia Sirlene Oliveira

**Affiliations:** 1 Instituto de Pesquisa Pelé Pequeno Príncipe, Curitiba 80250-060, Brazil; 2 Faculdades Pequeno Príncipe, Curitiba 80230-020, Brazil; 3 Curso de Medicina, Universidade Católica de Pelotas, Pelotas 96010-280, Brazil; qgarlet@gmail.com

**Keywords:** selenium, trace element, triple-negative breast cancer, cell line

## Abstract

Triple-negative breast cancer (TNBC) is an aggressive, fast-growing tumor that is more likely to spread to distant organs. Among women diagnosed with breast cancer, the prevalence of TNBC is 20%, and treatment is currently limited to chemotherapy. Selenium (Se), an essential micronutrient, has been explored as an antiproliferative agent. Therefore, this study aimed to evaluate the effects of exposure to organic (selenomethionine, ebselen, and diphenyl diselenide) and inorganic (sodium selenate and sodium selenite) Se molecules in different breast cell lines. The compounds were tested at 1, 10, 50, and 100 μM for 48 h in the non-tumor breast cell line (MCF-10A) and TNBC derivatives cell lines (BT-549 and MDA-MB-231). The effects of Se on cell viability, apoptotic and necrotic processes, colony formation, and cell migration were analyzed. Exposure to selenomethionine and selenate did not alter the evaluated parameters. However, selenomethionine had the highest selectivity index (SI). The exposure to the highest doses of selenite, ebselen, and diphenyl diselenide resulted in antiproliferative and antimetastatic effects. Selenite had a high SI to the BT cell line; however, the SI of ebselen and diphenyl diselenide was low in both tumoral cell lines. In conclusion, the Se compounds had different effects on the breast cell lines, and additional tests are needed to reveal the antiproliferative effects of Se compounds.

## 1. Introduction

Breast cancer (BC) is one of the most common types of cancer among women. According to the National Cancer Institute, in 2020, more than 66,000 new cases of breast cancer were diagnosed [1]. The World Health Organization estimates that by 2030, the population of women diagnosed with breast cancer could increase by up to 32% [2]. Breast cancer presents considerable heterogeneity and is classified according to its immunohistochemical profile as luminal A, luminal B, human epidermal growth factor receptor 2 (HER2-positive), triple-negative breast cancer (TNBC) or basal-like and recently discovered Claudin-low [3,4,5].

TNBC is a specific subtype of breast cancer that does not express estrogen, progesterone, and HER2 receptors. Clinical features of TNBC include high invasiveness, high metastatic potential, and poor prognosis [6]. TNBC represents 12–20% of all breast cancer diagnoses and is prevalent in premenopausal women under 40 years of age [7]. The treatment of TNBC is limited to a few therapeutic options. The major obstacle to the successful treatment of this disease is overcoming the lack of therapeutic targets owing to its negative profile, that is, the lack of estrogen, progesterone, and HER2 receptors expression [8,9,10]. 

Studies have been proposing the use of poly (ADP-ribose) polymerase inhibitors (PARPi) as a TNBC potential treatment; however, PARPi are reported to be effective in patients with BRCA1/2 germline mutations [11,12]. Engel et al. [13] observed that in a cohort of 802 women with TNBC, around 15% of the subjects had the BRCA 1/2 germline mutation. Thus, considering the mutations’ etiologies prevalence, the therapy with PARPi may not be effective in a large percentage of women [13]. In this context, the search for molecules that will induce cancer cells’ DNA damage with few side effects to the non-tumor cells is still pivotal. In breast cancer therapy, the study of trace elements is expanding intensively [14,15,16,17]. Recently, as studied by Pramanik et al. [18], copper-induced relevant antiproliferative effects in vitro (MDA-MB-231 cell line), particularly when inserted in lipid nanoparticles, which are pharmaceutical formulations designed to enclose chemotherapy drugs and deliver them more directly to the target cells.

Studies on selenium (Se) provide examples of scientific efforts to search for new options for cancer treatment [19,20,21]. Se is an essential dietary supplement, and it is incorporated as selenocysteine (Sec) into selenoproteins, some of which exist as antioxidant enzymes and are paramount for human health [22,23]. Preclinical studies observed the antiangiogenic, antiproliferative, and antimetastatic effects of several Se-containing molecules [24,25,26,27,28,29,30]. Moreover, some studies showed that the co-exposure of Se and chemotherapy drugs (e.g., paclitaxel and docetaxel) increases the antiproliferative effects of these drugs in TNBC cell lines [31,32]. The mechanisms and signaling pathways regulated by Se molecules are still under discussion [33,34]. Recently, Pan et al. [30] demonstrated that the antiproliferative mechanism of Se can occur by triggering oxidative or reductive stress depending on O_2_ presence. Therefore, this study aims to enrich the research on the anticancer effects of Se derivatives in TNBC cell lines. The chemical classes evaluated in this study were organic (selenomethionine), inorganic (selenate and selenite), and organoselenium (ebselen and diphenyl diselenide) compounds.

## 2. Materials and Methods

### 2.1. Breast Cell Lines

The MCF-10A (non-tumoral) and MDA-MB-231 (metastatic TNBC) cell lines were purchased from the cell bank of Rio de Janeiro, Brazil, and the BT-549 (primary TNBC) cell line was kindly provided by Dr. Luciane R. Cavalli from Instituto de Pesquisa Pelé Pequeno Príncipe, Brazil.

### 2.2. Cell Culture

Cells were cultured in Dulbecco’s Modified Eagle’s Medium/nutrient Mixture F-12 (DMEM-F12) (Sigma-Aldrich^®^—St. Louis, MO, USA), supplemented with 10% fetal bovine serum (FBS) (Sigma-Aldrich^®^—USA) and 1% penicillin/streptomycin (Sigma-Aldrich^®^—USA). The cell lines were maintained in a 75 cm^2^ culture flask at 37 °C in a humidified atmosphere with 5% CO_2_. The medium was replaced every 2–3 days.

### 2.3. Selenium Compounds

**Figure 1:**
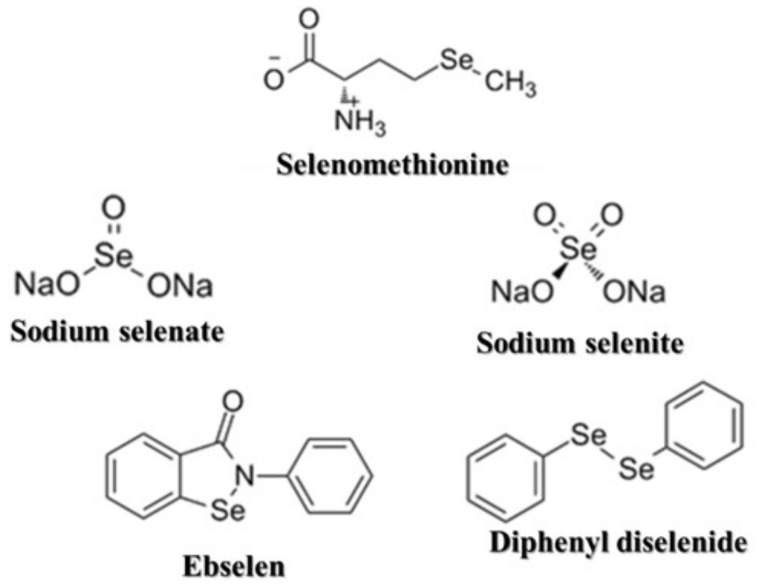
Selenium compounds tested in this work.

The Se compounds tested in this study included a naturally-derived organic molecule, selenomethionine (Cayman Chemical Company^®^—Ann Arbor, MI, USA), inorganic molecules sodium selenate (Sigma-Aldrich^®^—USA) and sodium selenite (Sigma-Aldrich^®^—USA), and synthetic organoselenium molecules, ebselen (Sigma-Aldrich^®^—USA) and diphenyl diselenide (Sigma-Aldrich^®^—USA) (Figure 1). Selenomethionine, sodium selenate, and sodium selenite were prepared in phosphate-buffered saline (PBS) (Sigma-Aldrich^®^—USA). Ebselen and diphenyl diselenide were prepared in dimethyl sulfoxide (DMSO) (Êxodo Cientifica^®^—Sumaré, Brazil).

### 2.4. Selenium Exposure 

Breast cells were plated into 6-, 24-, and 96-well plates at densities of 8 × 10^4^, 4 × 10^4^, or 1 × 10^4^ cells/well depending on the assay performed. After 24 h, the complete DMEM-F12 medium was removed and FBS-free DMEM-F12 was added to stabilize cell growth. For Se exposure, FBS-free DMEM-F12 was replaced with complete DMEM-F12 medium, and breast cells were exposed to 1, 10, 50, and 100 µM Se compounds for 48 h.

### 2.5. Cell Viability Assay 

After Se exposure, the cell medium was removed and replaced with a solution containing 3-(4,5-dimethylthiazol-2-yl)-2,5-diphenyltetrazolium bromide (MTT, 1 mg/mL of FBS-free DMEM-F12) (Invitrogen^®^—Waltham, MA, USA), and the plates were incubated at 37 °C. After 3 h of incubation, the supernatant was removed and 200 µL of DMSO was added to dissolve the formazan crystals. Absorbance was measured spectrophotometrically at a wavelength of 595 nm [35]. The results are expressed as percentages of the control.

### 2.6. Size and Granularity 

After Se exposure, the cell medium was collected, and the cells were washed twice with PBS and harvested using trypsin (Sigma-Aldrich^®^—USA). The cell suspension was centrifuged at 341× *g* and washed twice with PBS to remove the trypsin-containing complete medium. The cells were quantified using a FACS Canto II flow cytometer (Becton Dickinson—East Rutherford, NJ, USA). Analyses were performed using Flowing software version 2.5.0, on SSC (Side SCatter) and FSC (Forward SCatter) channels. The results are expressed as median fluorescence intensity (MFI) [36].

### 2.7. Identification of Apoptotic and Necrotic Cells 

After Se exposure, the cell medium was collected, and the cells were washed twice with PBS and harvested using trypsin. The cell suspensions were centrifuged at 341× *g* and washed twice with PBS to remove the trypsin-containing complete medium. After centrifugation, the cell pellets were resuspended in 300 µL of binding buffer (BD Biosciences^®^—San Jose, CA, USA), to which 3 µL of FITC-conjugated Annexin V (Invitrogen^®^—USA) and 5 µL of 7 aminoactinomycin D (Invitrogen^®^—USA) were added, and the cell suspensions were incubated in the dark for 15 min. A FACS Canto II flow cytometer (Becton Dickinson—USA) with FITC and PERCP channels was used to evaluate the cells. Analyses were performed using Flowing software version 2.5.0. The results are expressed as percentages of control [37].

### 2.8. Colony Formation Assay 

After Se exposure, the cell medium was collected, and the cells were washed twice with PBS and harvested using trypsin. Cells were counted in a Neubauer chamber. Ninety cells from each treatment group were plated in 6-well plates for 14 days. The cells were then fixed with cold ethanol (70%) for 5 min and stained with 25% crystal violet solution [38]. The results were expressed as the percentage of cells able to form colonies (CAFC).

### 2.9. Cell Migration Assay

The cell migration test was performed using the scrape assay [39]. Cells were plated in a 6-well plate at a density of 4 × 10^4^ cells/well. After 24 h, the cells were exposed to a 2 h pre-treatment with mitomycin C (Sigma-Aldrich^®^—USA) to stop cell proliferation. Next, the cell monolayer was scratched (creating a cell-free gap) with a sterile cell comb, the supernatant medium was carefully removed, the cells were washed with PBS, and fresh complete DMEM-F12 medium and Se compounds (1 µM) were added. The scratch area was photographed using an inverted microscope (10 × magnification; Evos XL Core) before Se addition (time 0) and 24 h after Se exposure. The analyses were performed using the ImageJ Exe^®^ program. The results were expressed as the percentage of closure.

### 2.10. Statistical Analyses

At least three independent experiments were performed for all tests. The data were statistically analyzed using Prisma GraphPad software, version 5.0, using the Kruskal–Wallis test followed by Dunn’s test, and presented as median ± interquartile range or one-way ANOVA followed by Dunnett’s test and presented as mean ± SEM. Results were considered statistically significant at *p* < 0.05. Since the concentrations were not in a logarithmic scale, the IC_50_ values were determined by the linear regression using the best-fit values method and expressed as mean ± standard error mean (SEM). The equation that described the activity of each compound and cell line is presented in Table S1 of supplementary material. The selective index (SI) was calculated as a result of the relation IC_50NTC_/IC_50TC_, where IC_50NTC_ is the concentration that decreased non-tumoral cells to 50% of the original count and IC_50TC_ is the concentration that decreased tumoral cell lines to 50% of the original count. The SI was calculated for each component tested in the different cell lines analyzed.

## 3. Results

### 3.1. Cell Viability

**Figure 2:**
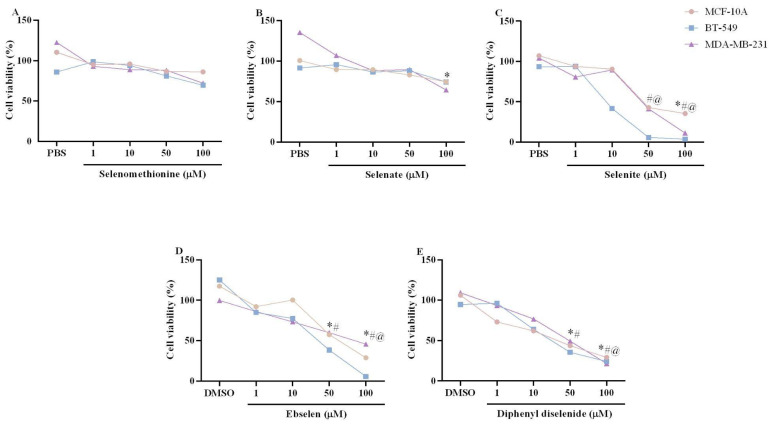
Cell viability analysis of non-tumoral breast cell line (MCF-10A) and tumoral breast cell lines (BT-549 and MDAMB-231) exposed for 48 h to selenomethionine (**A**), selenate (**B**), selenite (**C**), ebselen (**D**), and diphenyl diselenide (**E**). The results were analyzed by the Kruskal–Wallis test followed by Dunn’s post-test and presented as median (*n* = 4–6). The results presented as median ± interquartile range (*n* = 4–6) are presented in the supplementary material (Figure S1A–O of supplementary material). Symbols (* MCF-10A, ^@^ BT-549, and ^#^ MDA-MB-231) mean statistically significant differences from vehicle (PBS or DMSO). PBS: vehicle of selenomethionine, selenate, and selenite. DMSO: vehicle of ebselen and diphenyl diselenide.

The viability of breast cell lines exposed to Se compounds is shown in Figure 2A–E. The Kruskal–Wallis test revealed an absence of selenomethionine effects, in the tested concentrations, on cell viability in the three breast cell lines tested (Figure 2A). The IC_50_ values (µM) for the antiproliferative effect of selenomethionine in MCF-10 (441.76 ± 901.80), BT-549 (173.07 ± 1112.86), and MDA-MB-231 (197.66 ± 257.09) are shown in Table 1. The SI of selenomethionine was higher in BT-549 (2.55) than in MDA-MB-231 (2.23) cells (Table 1). The Kruskal–Wallis test showed an effect of selenate exposure on MCF-10A (H(5) = 10.03; *p* = 0.0399) and MDA-MB-231 (H(5) = 10.42; *p* = 0.0399) cell lines (Figure 2B). Exposure to 100 µM selenate caused a statistically significant decrease in the viability of MCF-10A and MDA-MB-231 cells. The IC_50_ values (µM) for the antiproliferative effect of selenate in MCF-10 (209.92 ± 614.78), BT-549 (246.04 ± 995.37), and MDA-MB-231 (187.54 ± 214.33) are shown in Table 1. The SI of selenate was higher in MDA-MB-231 (1.11) than in BT-549 (0.85) cells (Table 1). The Kruskal–Wallis test showed an effect of selenite exposure on MCF10-A (H(5) = 12.97; *p* = 0.0114), BT-549 (H(5) = 16.64; *p* = 0.0023), and MDA-MB-231 (H(5) = 13.62; *p* = 0.0086) cell lines (Figure 2C). Exposure to selenite caused a statistically significant decrease in cell viability of the MCF-10A cell line at 100 µM, the BT-549 cell line at 50 and 100 µM, and the MDA-MB-231 cell line at 100 µM. The IC_50_ values (µM) for the antiproliferative effect of selenite in MCF-10 (66.18 ± 268.88), BT-549 (29.54 ± 107.57), and MDA-MB-231 (50.04 ± 334.69) are shown in Table 1. The SI of selenite was higher in BT-549 (2.24) than in MDA-MB-231 (1.32) cells (Table 1). Regarding synthetic organoselenium compounds, the Kruskal–Wallis test showed an effect of ebselen (MCF-10A: H(5) = 14.31; *p* = 0.0064; BT-549: H(5) = 16.51; *p* = 0.0024; MDAMB-231: H(5) = 10.89; *p* = 0.0278) (Figure 2D) and diphenyl diselenide exposure (MCF-10A: H(5) = 17.91; *p* = 0.0013; BT-549: H(5) = 20.42; *p* = 0.0004; MDAMB-231: H(5) = 14.71; *p* = 0.0053) (Figure 2E) on the viability of the three breast cell lines tested. Ebselen and diphenyl diselenide exposure caused a statistically significant decrease in the viability of MCF-10A and BT-549 cell lines at 50 and 100 µM and of the MDA-MB-231 cell line at 100 µM. The IC_50_ values (µM) for the antiproliferative effect of synthetic organoselenium compounds in MCF-10 (ebselen: 82.07 ± 294.61; diphenyl diselenide: 56.86 ± 357.65), BT-549 (ebselen: 53.21 ± 346.94; diphenyl diselenide: 50.52 ± 483.46), and MDA-MB-231 (ebselen: 62.52 ± 374.96; diphenyl diselenide: 60.79 ± 242.19) are shown in Table 1. The SI was higher in BT-549 (ebselen: 1.54; diphenyl diselenide: 1.12) than in MDA-MB-231 (ebselen: 1.31; diphenyl diselenide: 0.93).

### 3.2. Size and Granularity

The size of the breast cell lines exposed to Se compounds is shown in Figure S2A–O of supplementary material. One-way ANOVA showed an absence of selenomethionine and selenite effects on cell size in the three breast cell lines tested, selenate and ebselen in BT-549 and MDA-MB-231 cells, and diphenyl diselenide in the MDA-MB-231 cell line. One-way ANOVA showed the effects of selenate [F(4,15) = 4.462; *p* = 0.0142], ebselen [F(4,14) = 14.84; *p* < 0.0001], and diphenyl diselenide [F(4,15) = 8.343; *p* = 0.0009] on the size of MCF-10A cells. In fact, MCF-10A cells exposed to 100 µM selenate and ebselen showed a statistically significant increase in cell size. In contrast, MCF-10A cells exposed to 50 and 100 µM diphenyl diselenide compounds showed a statistically significant decrease in cell size. Interestingly, one-way ANOVA showed an effect of diphenyl diselenide exposure on BT-549 cell size (F(4,10) = 3.872; *p* = 0.0376). Notably, BT-549 cells exposed to all concentrations of diphenyl diselenide showed a statistically significant decrease in size. The granularity of breast cell lines exposed to Se compounds is shown in Figure S3A–O of the supplementary material. One-way ANOVA showed an absence of selenomethionine, selenite, ebselen, and diphenyl diselenide effects on cell granularity in the three breast cell lines tested. In contrast, one-way ANOVA also revealed an effect of selenate [F(4,10) = 8.968; *p* = 0.0024] exposure on the granularity of MCF-10A cells. Exposure to 50 and 100 µM selenate caused a statistically significant increase in cell granularity.

### 3.3. Identification of Apoptotic and Necrotic Cells

**Figure 3:**
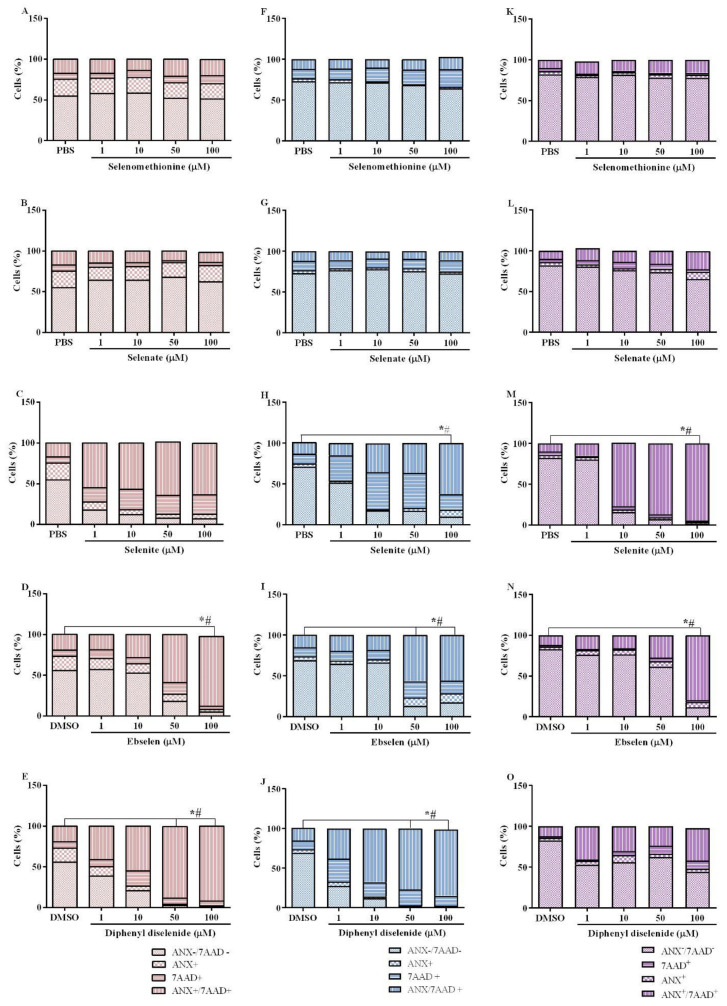
Identification of cell death by necrosis and apoptosis of non-tumor breast cell line (MCF-10A) and breast tumor cell lines (BT-549 and MDA-MB-231) exposed for 48 h to selenomethionine (**A**,**F**,**K**), selenate (**B**,**G**,**L**), selenite (**C**,**H**,**M**), ebselen (**D**,**I**,**N**) and diphenyl diselenide (**E**,**J**,**O**). The results were analyzed using the Kruskal–Wallis test followed by Dunn’s post-test. Representative figure of an experiment. Results presented as median ± interquartile range (*n* = 3–4) are presented in Figures S4–S6 of supplementary material. “*” means statistically significant differences from vehicle (PBS or DMSO) in relation to viable cells (ANX-/7AAD-) and “#” means statistically significant differences from vehicle (PBS or DMSO) in relation to late apoptotic and/or necrotic cells (ANX+/7AAD+). PBS: selenomethionine, selenate, and selenite vehicle. DMSO: ebselen and diphenyl diselenide vehicle.

The identification of apoptotic and necrotic cells in breast cell lines exposed to Se compounds is shown in Figure 3A–O. The Kruskal–Wallis test revealed the absence of selenomethionine and selenate effects in the three cell lines evaluated. In contrast, the Kruskal–Wallis test revealed an effect of selenite exposure on the percentage of viable (ANX-/7AAD-) and late apoptotic and/or necrotic (ANX+/7AAD+) in BT-549 (ANX-/7AAD-: H(5) = 12.57; *p* < 0.0001; ANX+/7AAD+: H(5) = 9.600; *p* = 0.0176) and MDA-MB-231 (ANX-/7AAD-: H(5) = 11.77; *p* = 0.0008; ANX+/7AAD+: H(5) = 11.37; *p* = 0.0017) cells. Exposure to 100 µM selenite caused a significant decrease in the percentage of viable BT-549 and MDA-MB-231 cells and an increase in the percentage of late apoptotic and/or necrotic cells. The Kruskal–Wallis test revealed an effect of ebselen exposure on the percentage of viable and late apoptotic and/or necrotic MCF-10A (ANX-/7AAD-: H(5) = 9.467; *p* = 0.0159; ANX+/7AAD+: H(5) = 11.33; *p* = 0.0018), BT-549 (ANX-/7AAD-: H(5) = 11.23; *p* = 0.0022; ANX+/7AAD+: H(5) = 10.50; *p* = 0.0068), and MDA-MB-231 (ANX-/7AAD-: H(5) = 10.50; *p* = 0.0068; ANX+/7AAD+: H(5) = 10.27; *p* < 0.0090) cells. Exposure to 100 µM ebselen caused a statistically significant decrease in the percentage of viable MCF-10A, BT-549, and MDA-MB-231 cells, and an increase in the percentage of late apoptotic and/or necrotic cells. Regarding diphenyl diselenide exposure, the Kruskal–Wallis test revealed an effect of this compound on the percentage of viable cells and late apoptotic and/or necrotic MCF-10A (ANX-/7AAD-: H(5) = 12.44; *p* = 0.0143; ANX+/7AAD+: H(5) = 15.50; *p* < 0.0038) and BT-549 (ANX-/7AAD-: H(5) = 12.57; *p* < 0.0001; ANX+/7AAD+: H(5) = 10.90; *p* = 0.0038) cells. Notably, exposure to 50 and 100 µM ebselen caused a significant decrease in the percentage of viable MCF-10A and BT-549 cells and an increase in late apoptotic and/or necrotic cells.

### 3.4. Colony Formation Assay

**Figure 4:**
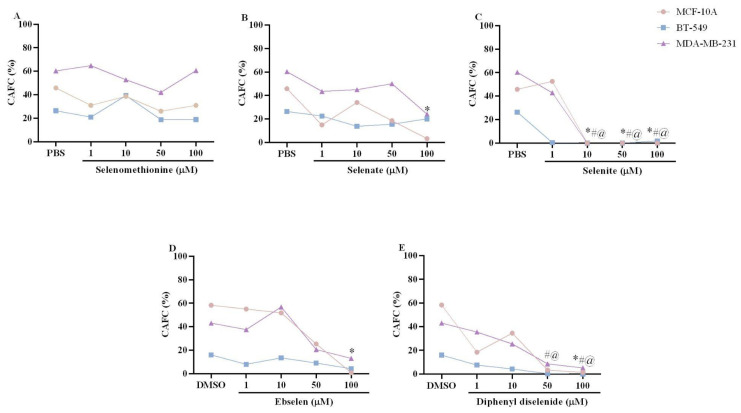
Colony forming unit of non-tumoral breast cell line (MCF-10A) and tumoral breast cell line (BT-549 and MDA-MB-231) exposed for 48 h to selenomethionine (**A**), selenate (**B**), selenite (**C**), ebselen (**D**), and diphenyl diselenide (**E**). The results were analyzed by the Kruskal–Wallis test followed by Dunn’s post-test and presented as median (*n* = 4–6). The results presented as median ± interquartile range (*n* = 4–6) are in Figure S7A–O of the supplementary material. Symbols (* MCF-10A, ^@^ BT-549, and ^#^ MDA-MB-231) mean statistically significant differences from vehicle (PBS or DMSO). PBS: vehicle of selenomethionine, selenate, and selenite. DMSO: vehicle of ebselen and diphenyl diselenide.

The percentage of colony-forming units of the three breast cell lines exposed to Se compounds is shown in Figure 4A–E. The Kruskal–Wallis test revealed no effects of exposure to selenomethionine in the three breast cell lines evaluated in this study. However, the Kruskal–Wallis test also showed an effect of selenate (H(5) = 10.44; *p* = 0.0336) and ebselen (H(5) = 10.69; *p* = 0.0302) exposure on the MCF-10A cell line. Specifically, MCF-10A cells exposed to 100 µM selenate or ebselen showed a significant decrease in the percentage of colonies formed. Furthermore, the Kruskal–Wallis test also showed an effect of selenite (MCF-10A: H(5) = 14.54; *p* = 0.0058; BT-549: H(5) = 15.49; *p* = 0.0038; MDA-MB-231: H(5) = 15.76; *p* = 0.0034) and diphenyl diselenide (MCF-10A: H(5) = 10.30; *p* = 0.0099; BT-549: H(5) = 13.64; *p* = 0.0086; MDA-MB-231: H(5) = 15.05; *p* = 0.0046) exposure on the three breast cell lines evaluated. Interestingly, exposure to 10–100 µM selenite completely inhibited the colony formation ability of MCF-10A, BT-549, and MDA-MB-231 cells. Exposure to 100 µM diphenyl diselenide also caused a statistically significant decrease in the percentage of MCF-10A cell colonies formed, and exposure to 50 and 100 µM diphenyl diselenide caused a statistically significant decrease in the percentage of colonies formed by BT-549 and MDA-MB-231 cells.

### 3.5. Cell Migration

**Figure 5:**
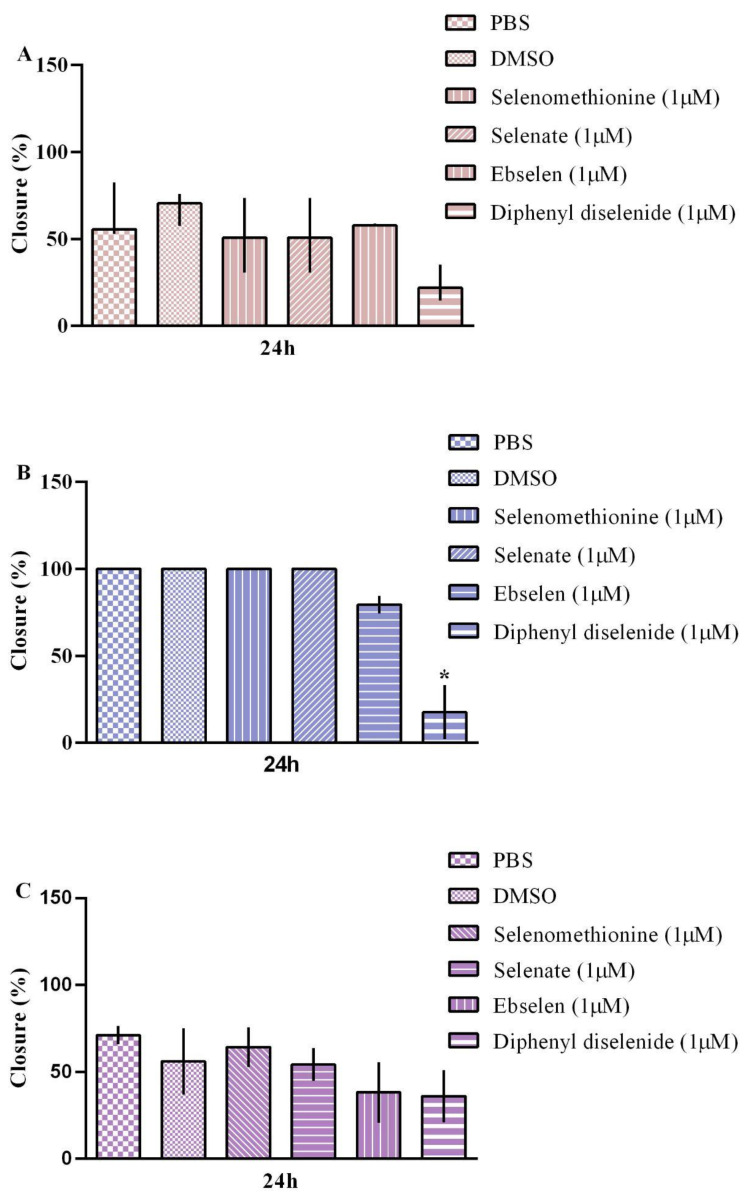
Cell migration of (**A**) MCF-10A (non-tumoral breast cell line), (**B**) BT-549 (tumoral breast cell line), and (**C**) MDAMB-231 (tumoral breast cell line) exposed for 48 h to selenomethionine, selenate, selenite, ebselen, and diphenyl diselenide. The results were analyzed by the Kruskal–Wallis test followed by Dunn’s post-test and presented as median ± interquartile range (*n* = 3–6). “*” means statistically different from the control vehicle. PBS: vehicle of selenomethionine, selenate, and selenite. DMSO: vehicle of ebselen and diphenyl diselenide.

The migration of breast cell lines exposed to Se compounds is shown in Figure 5A–C. The Kruskal–Wallis test revealed an absence of treatment effects on MCF-10A and MDA-MB-231 cell migration. In contrast, the Kruskal–Wallis test showed an effect of diphenyl diselenide exposure on BT-549 (H(3): 7.448; *p* = 0.0036) migration. In fact, exposure to 1 µM diphenyl diselenide significantly decreased BT-549 cell migration compared to the vehicle control (DMSO). Interestingly, exposure to 1 µM selenite caused cell detachment. Thus, it was impossible to evaluate the cell migration ability of the three breast cell lines evaluated in this study (Figures S8–S10 of supplementary material).

## 4. Discussion

Since the discovery of cancer, researchers worldwide have been studying new treatments that are less aggressive to healthy cells in the body. Treatment with Se and its derivatives has gained strength over the years, especially as dietary supplements for cancer prevention [40]. The results obtained in this study showed that different chemical forms of Se have different effects on mammary cell lines. 

Only 30% of women with TNBC are responsive to chemotherapy, meaning mortality rates remain high among these patients [41]. Thus, finding new molecules with high selectivity index toward cancer cells is crucial to improve the survival rates of patients diagnosed with a highly metastatic cancer, such as TNBC [42,43]. Using the MTT assay, a method used to evaluate viable cells with intact mitochondria [44], we observed that neither selenomethionine nor sodium selenate presented antiproliferative effects at the concentrations tested. Interestingly, selenomethionine had the highest IC_50_ in the three breast cell lines evaluated and had the highest SI, which suggests promising selectivity. Selenomethionine is the principal dietary source of Se and generally exerts dose-dependent chemoprevention effects without signs of toxicity [45,46]. In contrast, the exposure of breast cells to selenite, ebselen, and diphenyl diselenide decreased cell viability. Interestingly, diphenyl diselenide was cytotoxic to non-tumor and breast tumor cells, i.e., a similar percentage of viable cells was observed among the cell lines, as well as a low SI. Selenite is being studied as a putative anti-cancer molecule and its previously reported pharmacological effects include the inhibition of uncontrolled proliferation of cancer cells and decreased expression of MMP-9 adhesion protein, which in turn prevents metastases and increases cell death [30,47,48,49,50]. Organoselenium compounds are extensively studied due to their biological and redox modulation activities [51,52]. On the other hand, the prevention and treatment of cancer have been linked to their anti-inflammatory and antioxidant activities [53,54,55,56]. Our study explored the effects of two synthetic organoselenium compounds, ebselen and diphenyl diselenide, on tumoral and non-tumoral cells and detected that both compounds cause cell death and prevent the growth of human breast cancer. Additionally, we detected SI with translational potential to clinical results with some degree of safety. Therefore, further study of these molecules in vivo could provide a better understanding of this activity.

After exposure to Se compounds, cell death by necrosis and apoptosis was detected in all breast cell lines tested. In the BT-549 and MDA-MB-231 cell lines, selenite induced cell death at 100 µM. This observation confirms the findings of Kieliszek et al. [57], who observed that exposure to selenite prevented growth and induced apoptosis of cancer cells in vitro.

Cell migration is a fundamental process that allows the coordinated movement of a group of cells. Cells use focal adhesions, which are correction points in the extracellular matrix, for cell migration [58]. For metastatic processes, tumor cells must dissociate from neighboring cells, break the tight junction barrier, penetrate the vascular endothelium, migrate to other organs, and form new colonies [59]. The metastatic capacity of cells derived from solid tumors is a major cause of cancer-related mortality [60]. In our study, we observed that selenite and diphenyl diselenide reduced the ability of tumor cells to form colonies. In addition to preventing the formation of BT-549 cell line colonies, diphenyl diselenide inhibited cell migration. To the best of our knowledge, this is the first time that the antiproliferative and antimetastatic effects of Se compounds have been explored in the BT-549 mammary cell line, which is representative of primary TNBC.

## 5. Conclusions

The results indicate that at the concentrations tested, selenomethionine did not affect the mammary cell lines; however, the SI of selenomethionine to tumoral breast cell lines suggests an important specific antiproliferative potential of this molecule. Exposure to selenite resulted in cell death in tumor cells at concentrations of 50 and 100 µM and inhibition of colony formation at 10, 50, and 100 µM. Exposure to organoselenium compounds resulted in antiproliferative effects and inhibition of colony formation at the highest concentrations tested. We hope that this study will help the scientific community to better understand the effects of different chemical forms of selenium in breast cells. We consider that new tests with selenomethionine should be carried out in concentrations above 100 µM and selenite, ebselen, and diphenyl diselenide should be tested in concentrations below 10 µM.

## Figures and Tables

**Table 1:** The IC_50_ values (µM) and SI.

	MCF-10	BT-549		MDA-MB-231	
	IC_50_ (µM)	IC_50_ (µM)	SI	IC_50_ (µM)	SI
Selenomethionine	441.76 ± 901.80	173.07 ± 1112.86	2.55	197.66 ± 257.09	2.23
Selenate	209.92 ± 614.78	246.04 ± 995.37	0.85	187.54 ± 214.33	1.11
Selenite	66.18 ± 268.88	29.54 ± 107.57	2.24	50.04 ± 334.69	1.32
Ebselen	82.07 ± 294.61	53.21 ± 346.94	1.54	62.52 ± 374.96	1.31
Diphenyl diselenide	56.86 ± 357.65	50.52 ± 483.46	1.12	60.79 ± 242.19	0.93

The results are presented as mean ± SEM. SI = selectivity index.

## Data Availability

The data are available from the corresponding author.

## Supplementary Materials

The following supporting information can be downloaded at: https://www.mdpi.com/article/10.3390/biomedicines11051346/s1, Figure S1: Analysis of cell viability detected by the MTT (3-[4,5-dimethylthiazol-2-yl]-2,5 diphenyl tetrazolium bromide) assay in a non-tumor breast cell line (MCF-10A) and breast tumor cell lines of the triple-negative subtype (BT-549 and MDA-MB-231) exposed for 48 h to selenomethionine (**A**, **F**, and **K**), selenate (**B**, **G**, and **L**), selenite (**C**, **H**, and **M**), ebselen (**D**, **I**, and **N**) and diphenyl diselenide (**E**, **J**, and **O**); Figure S2: Size of non-tumor cell line (MCF-10A) and breast tumor cell lines of the triple-negative subtype (BT-549 and MDAMB-231) after exposure for 48 h to selenomethionine (**A**, **F**, and **K**), selenate (**B**, **G**, and **L**), selenite (**C**, **H**, and **M**), ebselen (**D**, **I**, and **N**) and diphenyl diselenide (**E**, **J**, and **O**); Figure S3: Granularity of non-tumor cell line (MCF-10A) and breast tumor cell lines of the triple-negative subtype (BT-549 and MDAMB-231 after exposure for 48 h to selenomethionine (**A**, **F**, and **K**), selenate (**B**, **G**, and **L**), selenite (**C**, **H**, and **M**), ebselen (**D**, **I**, and **N**) and diphenyl diselenide (**E**, **J**, and **O**); Figure S4: Percentage of viable (ANX-/7AAD-) and apoptotic or necrotic MCF-10A cells (ANX+/7AAD+) exposed for 48 h to selenomethionine (**A** and **F**), selenate (**B** and **G**), selenite (**C** and **H**), ebselen (**D** and **I**), and diphenyl diselenide (**E** and **J**); Figure S5: Percentage of viable (ANX-/7AAD-) and apoptotic or necrotic BT-549 cells (ANX+/7AAD+) exposed for 48 h to selenomethionine (**A** and **F**), selenate (**B** and **G**), selenite (**C** and **H**), ebselen (**D** and **I**), and diphenyl diselenide (**E** and **J**); Figure S6: Percentage of viable (ANX-/7AAD-) and apoptotic or necrotic MDA-MB-231 cells (ANX+/7AAD+) exposed for 48 h to selenomethionine (**A** and **F**), selenate (**B** and **G**), selenite (**C** and **H**), ebselen (**D** and **I**), and diphenyl diselenide (**E** and **J**); Figure S7: Colonies from non-tumor breast cell lines (MCF-10A) and triple-negative subtype breast tumor cell lines (BT-549 and MDA-MB-231) analyzed by crystal violet staining and exposed for 48 h to selenomethionine (**A**, **F**, and **K**), selenate (**B**, **G**, and **L**), selenite (**C**, **H**, and **M**), ebselen (**D**, **I**, and **N**) and diphenyl diselenide (**E**, **J**, and **O**); Figure S8: Cell migration. Photographs were taken under an inverted microscope (10× magnification; Evos XL Core) before the addition of Se compounds (time 0) and after exposure to Se (time 24 h) of non-tumor breast cell lines (MCF-10A); Figure S9: Cell migration. Photographs were taken under an inverted microscope (10× magnification; Evos XL Core) before the addition of Se compounds (time 0) and after exposure to Se (time 24 h) of non-tumor breast cell lines (BT-549); Figure S10: Cell migration. Photographs were taken under an inverted microscope (10× magnification; Evos XL Core) before the addition of Se compounds (time 0) and after exposure to Se (time 24 h) of non-tumor breast cell lines (MDA-MB-231). Table S1: Equation used to calculate the IC_50_ values.
