# Supplementary material for: Antiproliferative Effect of Inorganic and Organic Selenium Compounds in Breast Cell Lines
Source: Biomedicines. 2023 May 3;11(5):1346. doi: 10.3390/biomedicines11051346 (PMC10216490; doi:10.3390/biomedicines11051346)
Supplement: Supplementary file 1 [file biomedicines-11-01346-s001.zip › biomedicines-2287186-supplementary.pdf]

biomedicines

Supporting Material

Antiproliferative effect of inorganic and organic selenium compounds in breast cell lines

Nayara de Souza da Costa[1,2], Luíza Siqueira de Lima[1,2], Franciele Aparecida Mendes de Oliveira[1], Maria Eduarda de Andrade Galiciolli[1,2], Mariana Inocêncio Manzano[1,2], Quelen Iane Garlet[3], Ana Carolina Irioda[1,2*], Cláudia Sirlene Oliveira[1,2*]

[1]Instituto de Pesquisa Pelé Pequeno Príncipe, Curitiba, PR, Brazil.

[2]Faculdades Pequeno Príncipe, Curitiba, PR, Brazil.

[3]Curso de Medicina, Universidade Católica de Pelotas, RS, Brazil.

Table S1. Equation used to calculate the IC_{50} values.

	**MCF-10**	**BT-549**	**MDA-MB-231**
Selenomethionine	Y = -0.1123×X + 99.61	Y = -0.2139×X + 87.02	Y = -0.2445×X + 98.33
Selenate	Y = -0.2025×X + 92.51	Y = -0.1872×X + 96.06	Y = -0.2938×X + 105.1
Selenite	Y = -0.7032×X + 96.54	Y = -0.8767×X + 75.90	Y = -0.8592×X + 93.00
Ebselen	Y = -0.5780×X + 97.44	Y = -0.8749×X + 96.56	Y = -0.5030×X + 81.45
Diphenyl diselenide	Y = -0.6190×X + 85.20	Y = -0.7014×X + 85.44	Y = -0.7483×X + 95.49

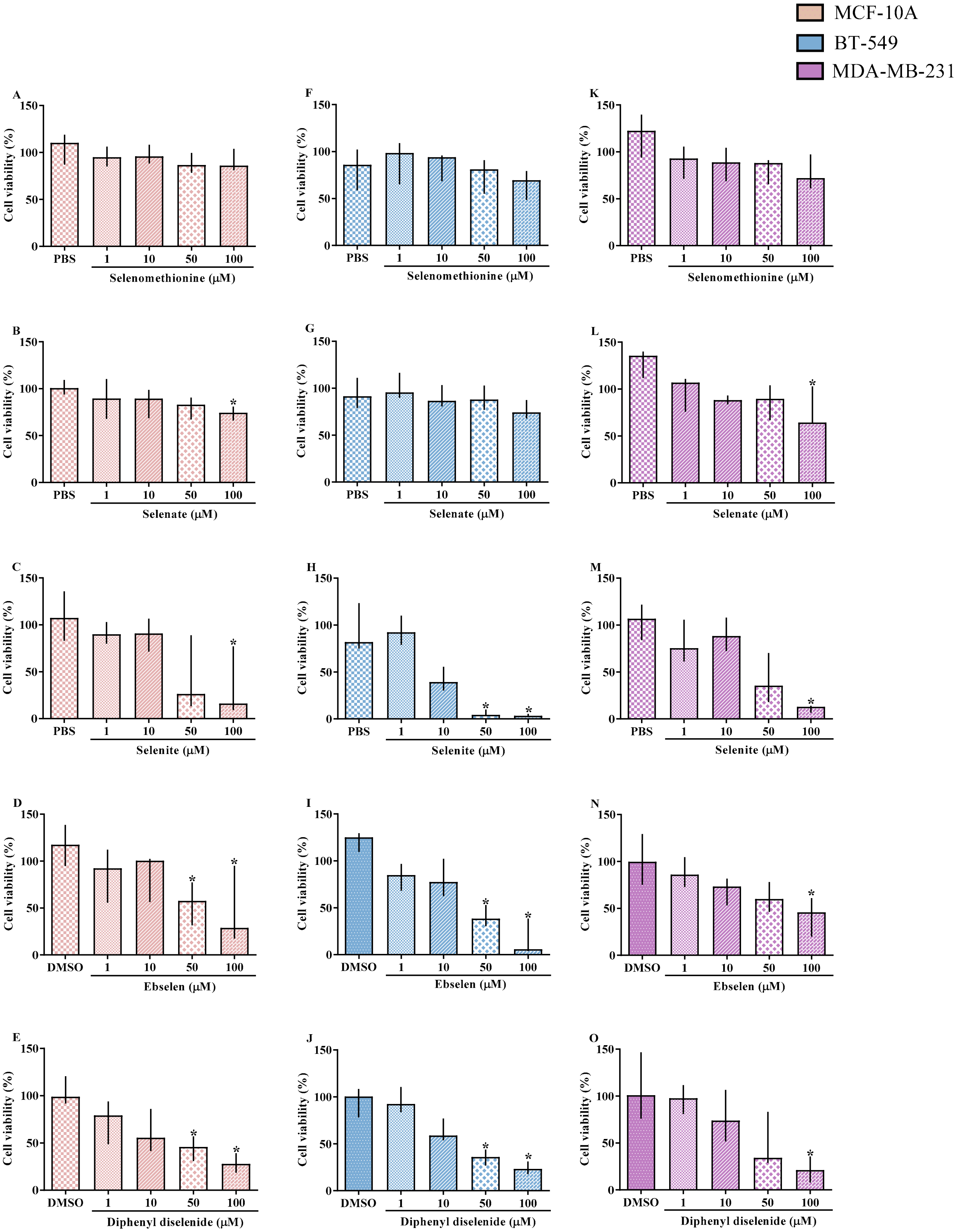

Figure S1. Analysis of cell viability detected by the MTT (3-[4,5-dimethylthiazol-2-yl]-2,5 diphenyl tetrazolium bromide) assay in a non-tumor breast cell line (MCF-10A) and breast tumor cell lines of the triple-negative subtype (BT-549 and MDA-MB-231) exposed

for 48h to selenomethionine (A, F, and K), selenate (B, G, and L), selenite (C, H, and M), ebselen (D, I, and N) and diphenyl diselenide (E, J, and O). The results were analyzed using the Kruskal-Wallis test followed by the Dunn post-test and presented as median ± interquartile range (n= 4-6). "*" means statistically different from the control vehicle. PBS: selenomethionine, selenate, and selenite vehicle. DMSO: ebselen and diphenyl diselenide vehicle.

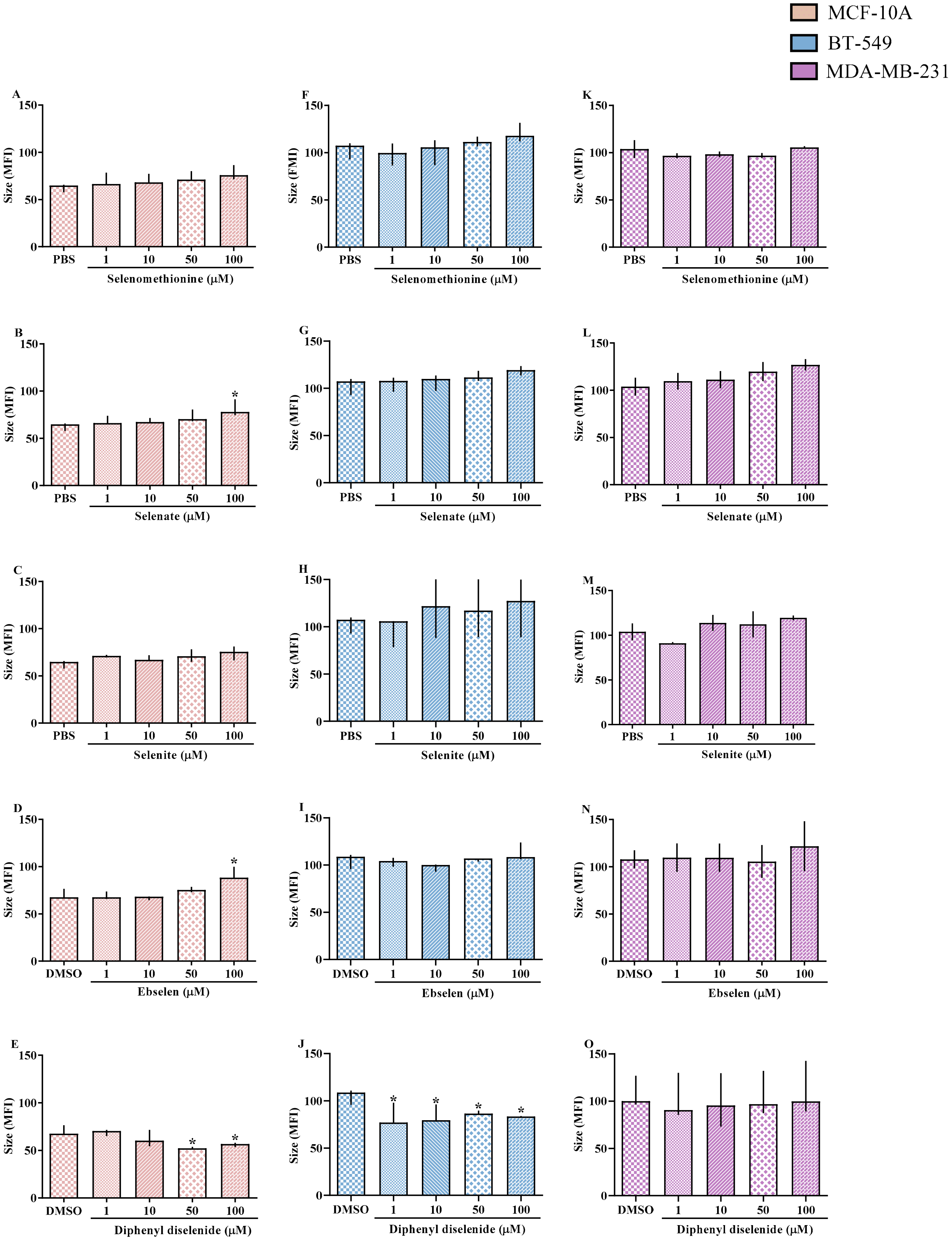
MCF-10A
BT-549
MDA-MB-231
A
F
K
B
G
L
C
H
M
D
I
N
E
J
O
Size (MFI)
Size (FMI)
150
100
50
0
PBS
DMSO
1
10
50
100
*
Selenomethionine (μM)
Selenate (μM)
Selenite (μM)
Ebselen (μM)
Diphenyl diselenide (μM)

Figure S2. Size of non-tumor cell line (MCF-10A) and breast tumor cell lines of the triple-negative subtype (BT-549 and MDAMB-231) after exposure for 48h to selenomethionine (A, F, and K), selenate (B, G, and L), selenite (C, H, and M), ebselen (D, I, and N) and diphenyl diselenide (E, J, and O). Results were analyzed by one-way ANOVA followed by Dunnett's post-test and presented as mean ± SEM (n= 3). "*" means statistically different from the control vehicle. PBS: selenomethionine, selenate, and selenite vehicle. DMSO: ebselen and diphenyl diselenide vehicle.

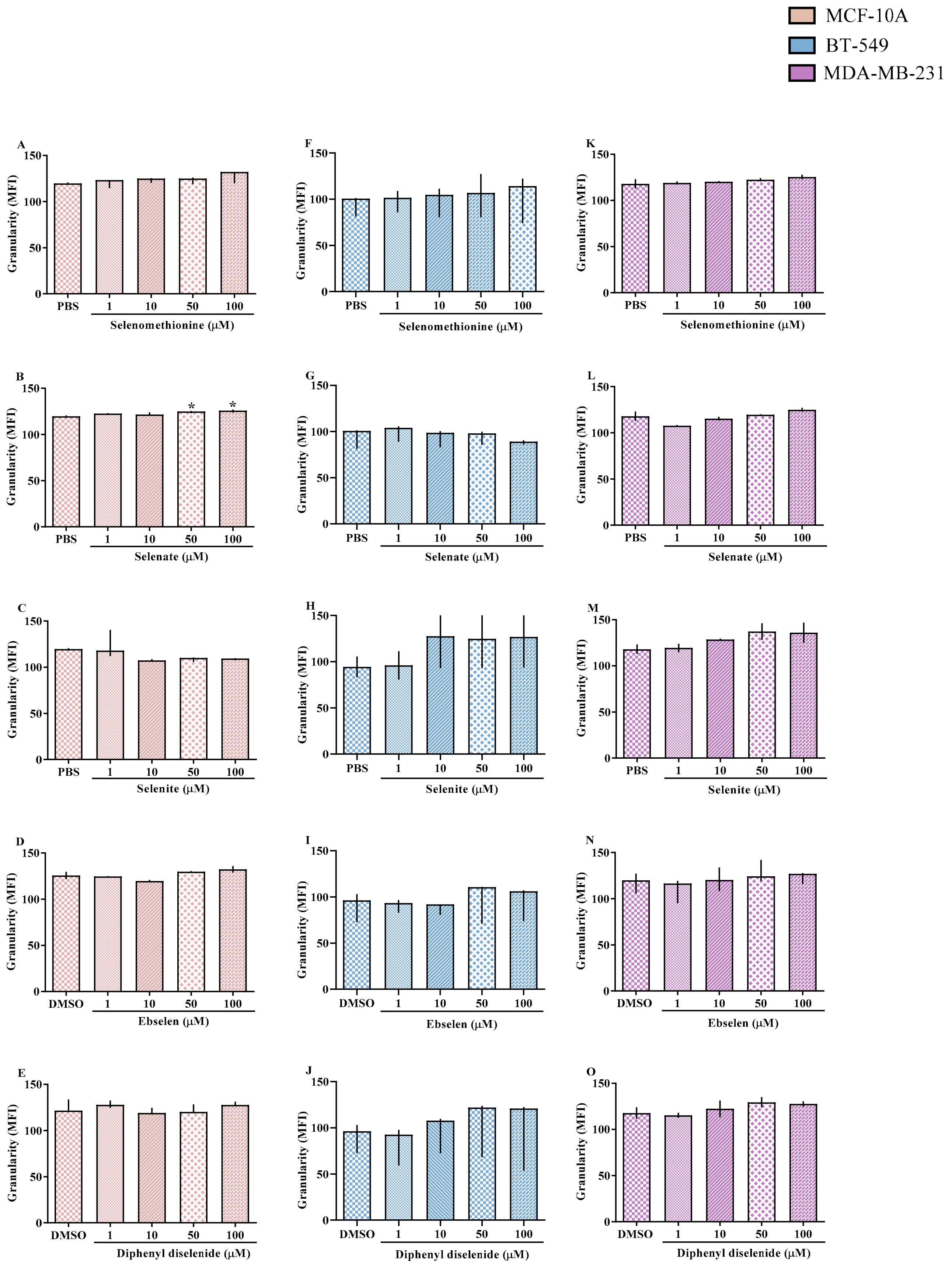
MCF-10A
BT-549
MDA-MB-231
A
F
K
B
G
L
C
H
M
D
I
N
E
J
O
Granularity (MFI)
0
50
100
150
PBS
DMSO
1
10
50
100
*
Selenomethionine (μM)
Selenate (μM)
Selenite (μM)
Ebselen (μM)
Diphenyl diselenide (μM)

Figure S3 of supplementary material: Granularity of non-tumor cell line (MCF-10A) and breast tumor cell lines of the triple-negative subtype (BT-549 and MDAMB-231 after exposure for 48h to selenomethionine (A, F, and K), selenate (B, G, and L), selenite (C, H, and M), ebselen (D, I, and N) and diphenyl diselenide (E, J, and O). Results were analyzed by one-way ANOVA followed by Dunnett's post-test and presented as mean ± SEM (n= 3). "*" means statistically different from the control vehicle. PBS: selenomethionine, selenate, and selenite vehicle. DMSO: ebselen and diphenyl diselenide vehicle.

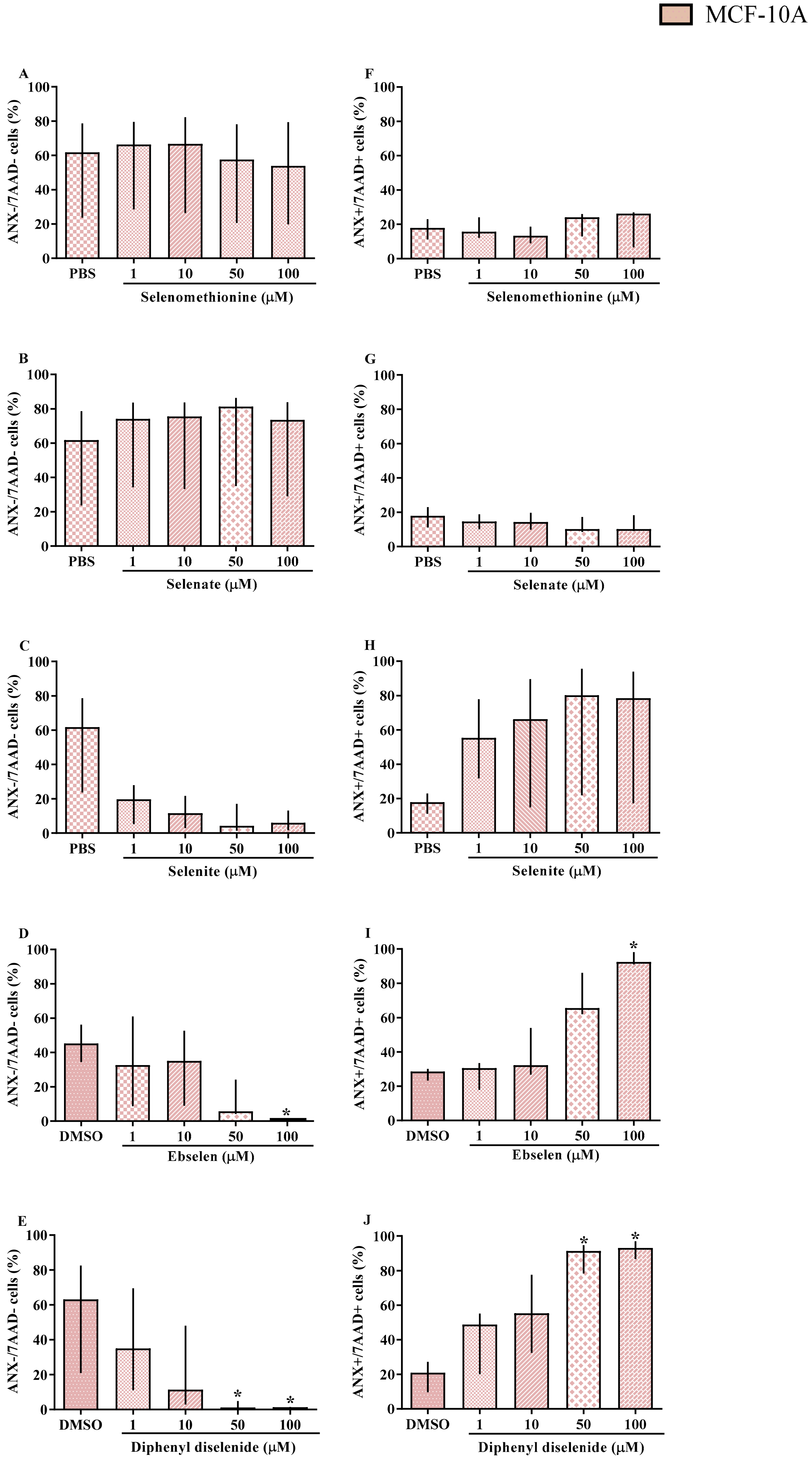
MCF-10A
A
ANX-/7AAD- cells (%)
PBS 1 10 50 100
Selenomethionine (μM)
F
ANX+/7AAD+ cells (%)
PBS 1 10 50 100
Selenomethionine (μM)
B
ANX-/7AAD- cells (%)
PBS 1 10 50 100
Selenate (μM)
G
ANX+/7AAD+ cells (%)
PBS 1 10 50 100
Selenate (μM)
C
ANX-/7AAD- cells (%)
PBS 1 10 50 100
Selenite (μM)
H
ANX+/7AAD+ cells (%)
PBS 1 10 50 100
Selenite (μM)
D
ANX-/7AAD- cells (%)
DMSO 1 10 50 100
Ebselen (μM)
I
ANX+/7AAD+ cells (%)
DMSO 1 10 50 100
Ebselen (μM)
E
ANX-/7AAD- cells (%)
DMSO 1 10 50 100
Diphenyl diselenide (μM)
J
ANX+/7AAD+ cells (%)
DMSO 1 10 50 100
Diphenyl diselenide (μM)

Figure S4 of supplementary material: Percentage of viable (ANX-/7AAD-) and apoptotic or necrotic MCF-10A cells (ANX+/7AAD+) exposed for 48 h to selenomethionine (A and F), selenate (B and G), selenite (C and H), ebselen (D and I), and diphenyl diselenide (E and J). The results were analyzed using the Kruskal-Wallis test followed by the Dunn post-test and presented as median ± interquartile range (n= 3-6). "*" means statistically different from control the control vehicle. PBS: selenomethionine, selenate, and selenite vehicle. DMSO: ebselen and diphenyl diselenide vehicle.

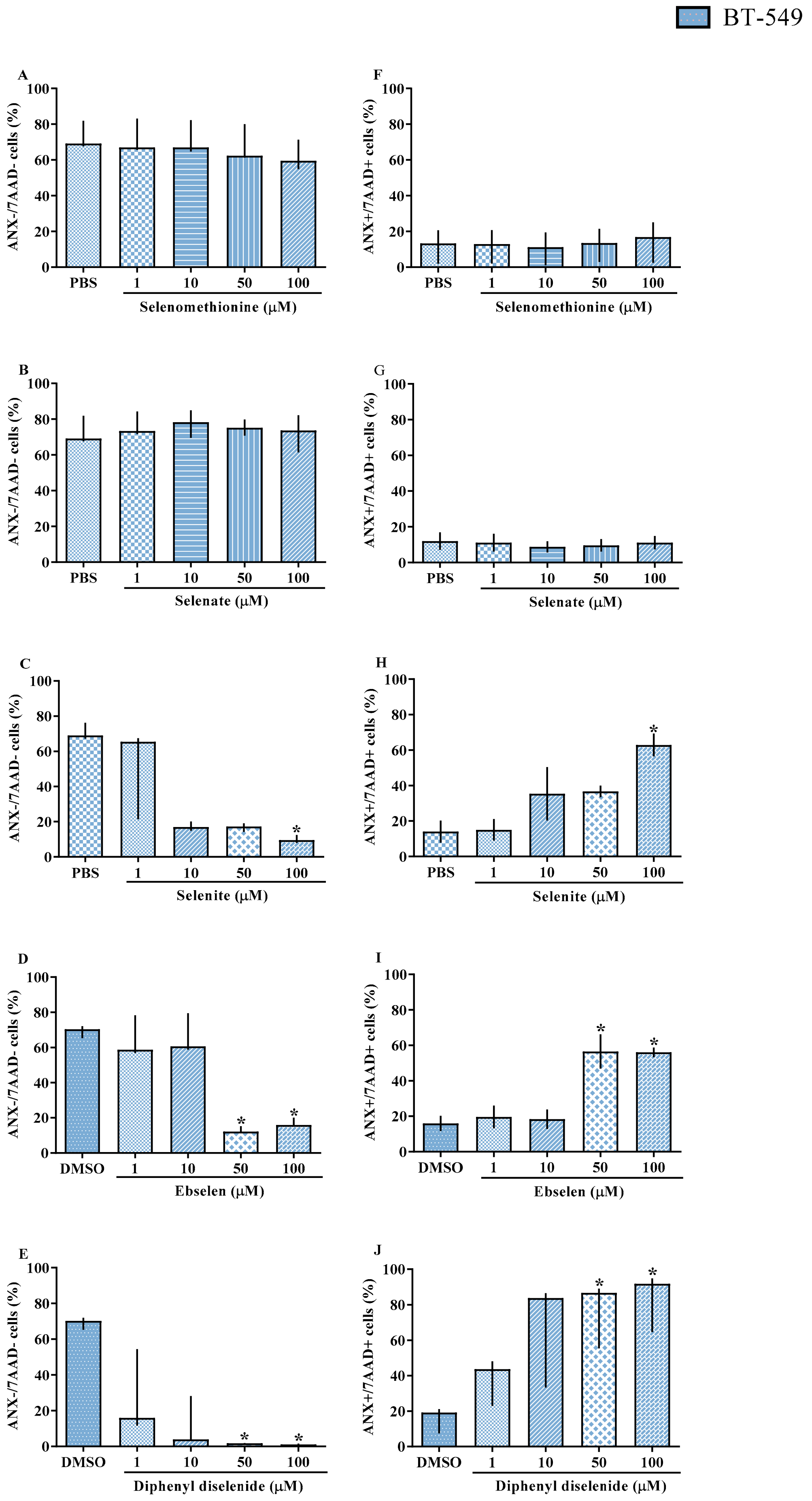
BT-549
A
ANX-/7AAD- cells (%)
PBS
1
10
50
100
Selenomethionine (μM)
F
ANX+/7AAD+ cells (%)
Selenomethionine (μM)
B
ANX-/7AAD- cells (%)
Selenate (μM)
G
ANX+/7AAD+ cells (%)
Selenate (μM)
C
ANX-/7AAD- cells (%)
Selenite (μM)
H
ANX+/7AAD+ cells (%)
Selenite (μM)
D
ANX-/7AAD- cells (%)
DMSO
Ebselen (μM)
I
ANX+/7AAD+ cells (%)
Ebselen (μM)
E
ANX-/7AAD- cells (%)
Diphenyl diselenide (μM)
J
ANX+/7AAD+ cells (%)
Diphenyl diselenide (μM)

Figure S5 of supplementary material: Percentage of viable (ANX-/7AAD-) and apoptotic or necrotic BT-549 cells (ANX+/7AAD+) exposed for 48 h to selenomethionine (A and F), selenate (B and G), selenite (C and H), ebselen (D and I), and diphenyl diselenide (E and J). The results were analyzed using the Kruskal-Wallis test followed by the Dunn post-test and presented as median ± interquartile range (n= 3-6). "*" means statistically different from control the control vehicle. PBS: selenomethionine, selenate, and selenite vehicle. DMSO: ebselen and diphenyl diselenide vehicle.

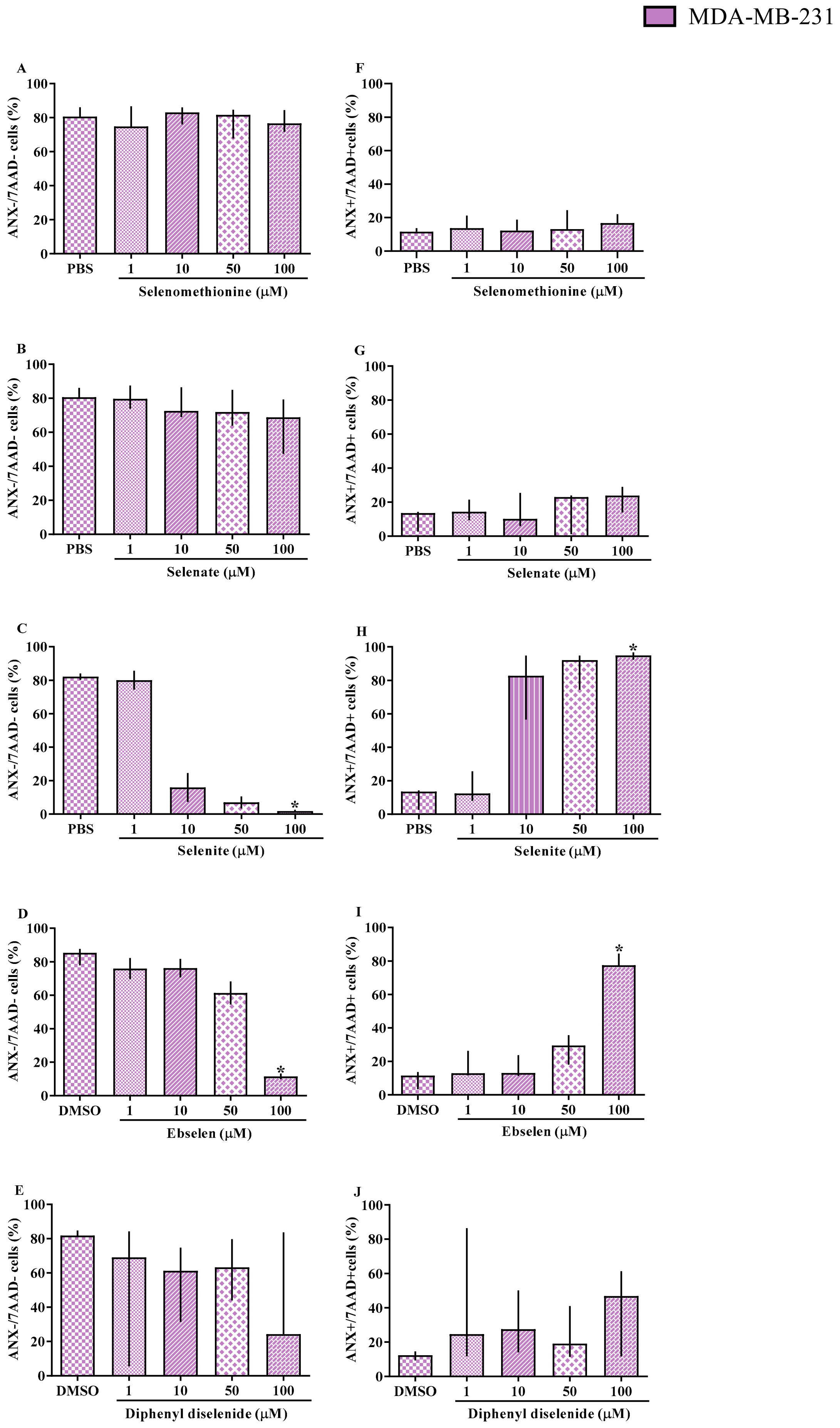
MDA-MB-231
A
ANX-/7AAD- cells (%)
PBS 1 10 50 100
Selenomethionine (μM)
F
ANX+/7AAD+cells (%)
PBS 1 10 50 100
Selenomethionine (μM)
B
ANX-/7AAD- cells (%)
PBS 1 10 50 100
Selenate (μM)
G
ANX+/7AAD+ cells (%)
PBS 1 10 50 100
Selenate (μM)
C
ANX-/7AAD- cells (%)
PBS 1 10 50 100
Selenite (μM)
H
ANX+/7AAD+ cells (%)
PBS 1 10 50 100
Selenite (μM)
D
ANX-/7AAD- cells (%)
DMSO 1 10 50 100
Ebselen (μM)
I
ANX+/7AAD+ cells (%)
DMSO 1 10 50 100
Ebselen (μM)
E
ANX-/7AAD- cells (%)
DMSO 1 10 50 100
Diphenyl diselenide (μM)
J
ANX+/7AAD+cells (%)
DMSO 1 10 50 100
Diphenyl diselenide (μM)
0 20 40 60 80 100
*

Figure S6 of supplementary material: Percentage of viable (ANX-/7AAD-) and apoptotic or necrotic MDA-MB-231 cells (ANX+/7AAD+) exposed for 48 h to selenomethionine (A and F), selenate (B and G), selenite (C and H), ebselen (D and I), and diphenyl diselenide (E and J). The results were analyzed using the Kruskal-Wallis test followed by the Dunn post-test and presented as median ± interquartile range (n= 3-6). "*" means statistically different from control the control vehicle. PBS: selenomethionine, selenate, and selenite vehicle. DMSO: ebselen and diphenyl diselenide vehicle.

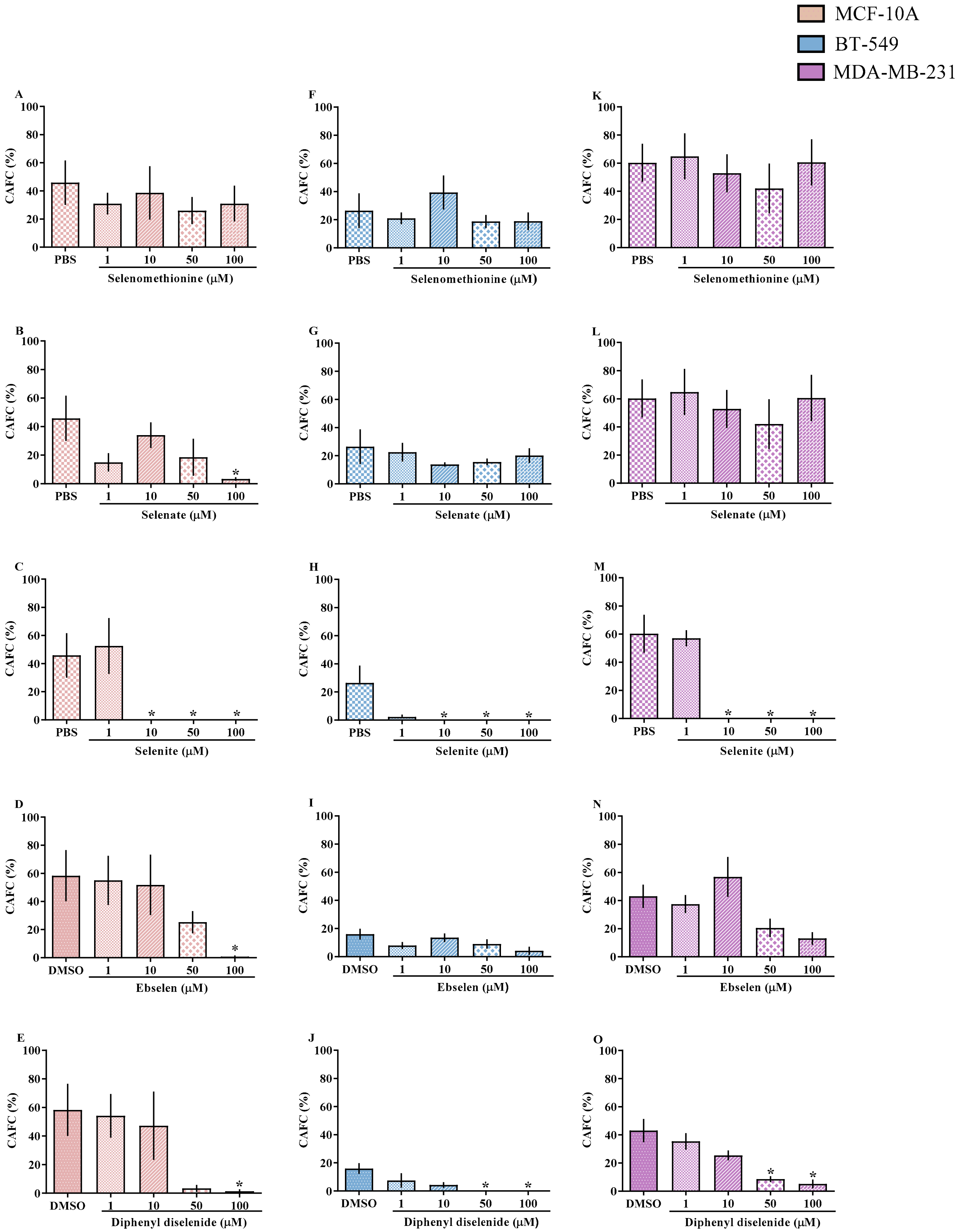
MCF-10A
BT-549
MDA-MB-231
A
F
K
CAFC (%)
PBS
1
10
50
100
Selenomethionine (μM)
B
G
L
Selenate (μM)
C
H
M
Selenite (μM)
D
I
N
DMSO
Ebselen (μM)
E
J
O
Diphenyl diselenide (μM)
*

Figure S7 of supplementary material: Colonies from non-tumor breast cell lines (MCF-10A) and triple-negative subtype breast tumor cell lines (BT-549 and MDA-MB-231) analyzed by crystal violet staining and exposed for 48h to selenomethionine (A, F, and K), selenate (B, G, and L), selenite (C, H, and M), ebselen (D, I, and N) and diphenyl diselenide (E, J, and O). The results were analyzed using the Kruskal-Wallis test followed by the Dunn post-test and presented as median ± interquartile range (n= 4-6). "*" means statistically different from the control vehicle. N.D.: not detected. PBS: selenomethionine, selenate, and selenite vehicle. DMSO: ebselen and diphenyl diselenide vehicle.

.

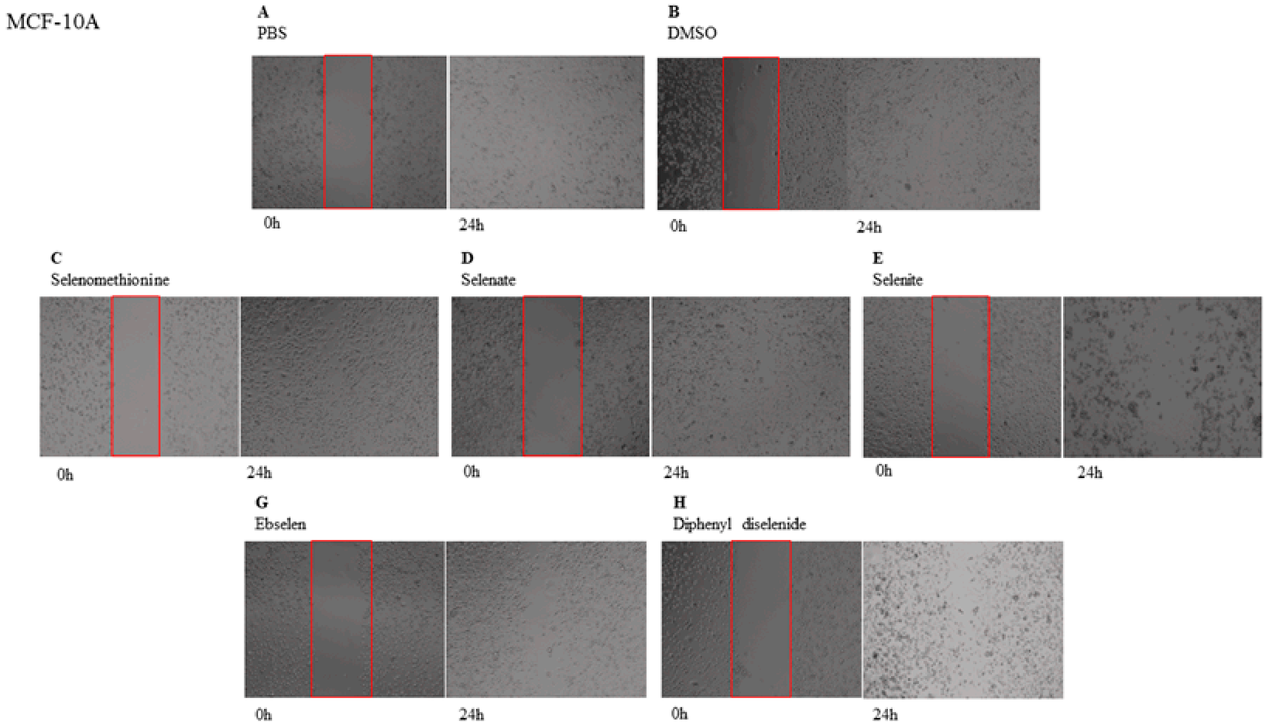

Figure S8 of supplementary material: Cell migration. Photographs were taken under an inverted microscope (10x magnification; Evos XL Core) before the addition of Se compounds (time 0) and after exposure to Se (time 24h) of non-tumor breast cell lines (MCF-10A). PBS: selenomethionine, selenate, and selenite vehicle. DMSO: ebselen and diphenyl diselenide vehicle.

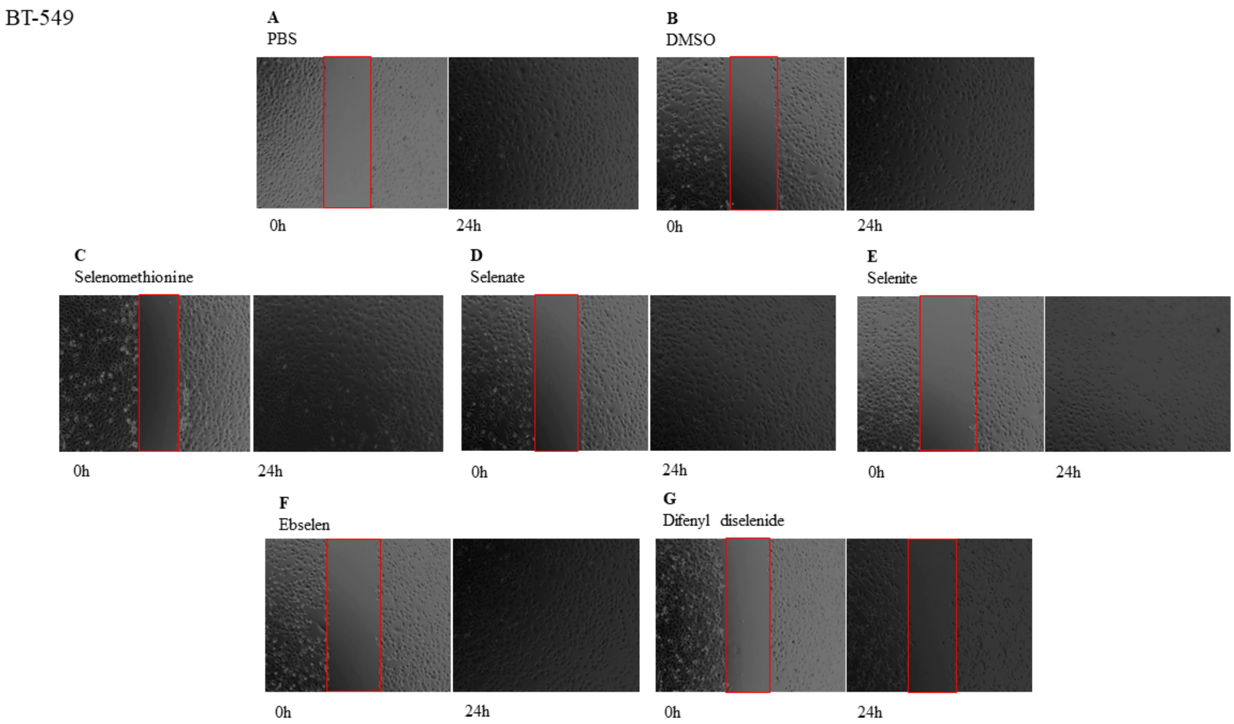

Figure S9 of supplementary material: Cell migration. Photographs were taken under an inverted microscope (10x magnification; Evos XL Core) before the addition of Se compounds (time 0) and after exposure to Se (time 24h) of non-tumor breast cell lines (BT-549). PBS: selenomethionine, selenate, and selenite vehicle. DMSO: ebselen and diphenyl diselenide vehicle.

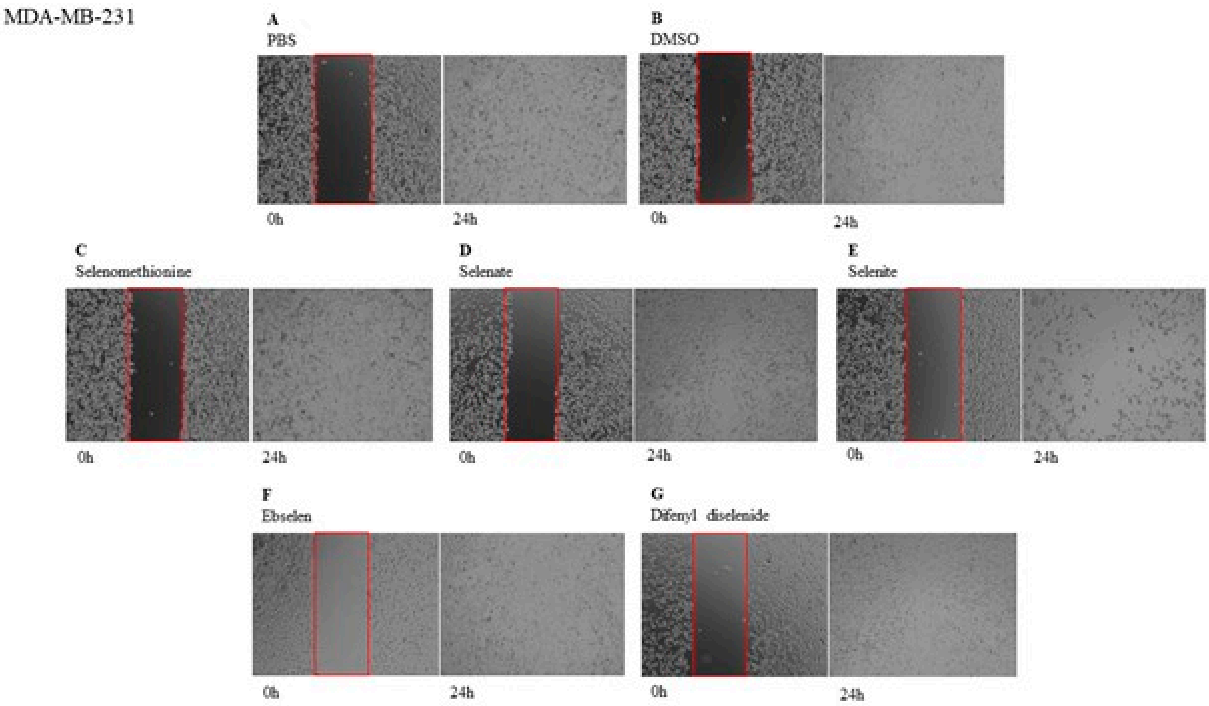

Figure S10 of supplementary material: Cell migration. Photographs were taken under an inverted microscope (10x magnification; Evos XL Core) before the addition of Se compounds (time 0) and after exposure to Se (time 24h) of non-tumor breast cell lines (MDA-MB-231). PBS: selenomethionine, selenate, and selenite vehicle. DMSO: ebselen and diphenyl diselenide vehicle.
